# Belladonna

**Published:** 1894-10

**Authors:** 


### HOMŒOPATHIC PHYSICIAN,

A MONTHLY JOURNAL OF

HOMŒOPATHIC MATERIA MEDICA AND CLINICAL MEDICINE.

"If our school ever gives up the strict inductive method of Hahnemann, we are lost, and deserve only to be mentioned as a caricature in the history of medicine."—CONSTANTINE HERING.

Vol. XIV. OCTOBER, 1894. No. 10.

### EDITORIAL.

BELLADONNA.—In the editorial of last month, concerning the use of Aconite, there was given a comparison with Belladonna. As Aconite and Belladonna are in some respects similar remedies belonging to the same group, it is well to know what the differences are between them, that each may be used only when truly indicated, and not confounded the one with the other, as is so commonly done by loose prescribers.

These differences are well marked and easily remembered, and should be committed to memory by every physician.

The red face of Belladonna, as previously stated, is a dark or purple redness, while the Aconite patient has a bright red face. The red face of the Belladonna patient is worse from leaning the head forward. In the Aconite patient, on the other hand, *the red face becomes pale if the head be raised from the pillow.* This is a great characteristic—a key-note—of Aconite, though Veratrum has the same symptom. The Belladonna patient *sees fantastic illusions and visions when he closes his eyes.* Here is a key-note of Belladonna which ought to be carefully remembered. Belladonna has aggravation, therefore, from closing the eyes, whilst Aconite is *better* from closing them.

Belladonna has headache on the right side of the head, while Aconite has it on the left side.

Belladonna is better from sitting up during the headache; lying down increases the headache.

Aconite, on the other hand, is better from lying down.

The Aconite patient has hot head, hands, and feet.

The Belladonna patient has *hot head and cold hands and feet.* Here again is a characteristic which should always be remembered when prescribing.

Belladonna has *beads of hot perspiration standing on the forehead,* whilst the body is at the same time burning hot.

Aconite has *hot, dry skin.*

The perspiration of Belladonna is apt to be pungent smelling, whilst Aconite has no perspiration until the paroxysm is over, and then it smells sour.

Generally speaking, Aconite is a left-sided remedy, while the affections of Belladonna are on the right side.

Belladonna affects the upper left and lower right side, while Aconite has the reverse.

Aconite has dryness of the mouth with thirst, while Belladonna has dryness *without* thirst.

Belladonna has chills descending, whilst with Aconite they ascend.

Belladonna has sleeplessness before midnight, Aconite after midnight.

Having, in the foregoing observations, shown some of the contrasts of Aconite and Belladonna, it may not be amiss to recount some of the special characteristics of Belladonna.

Belladonna takes cold from uncovering the head; therefore, from having the hair cut in cool weather.

The great characteristic of its pains is throbbing. Even when there is no pain, the patient can hear and feel throbbing all over him when lying in bed, especially in the morning.

The patient is *sensitive to the jarring of the bed on which he lies.* This is one of the surest indications for Belladonna in the whole range of its pathogenesis. The editor has repeatedly verified it. One of the most remarkable cures which he ever made was by paying attention to this symptom.

A young man who had contracted an attack of gonorrhœa had consulted a physician of the old school of medicine, who prescribed astringent injections which speedily dried up the flow.

But an attack of acute inflammation of the prostate gland followed for which Opium suppositories were applied with leeches to the perineum. The patient became rapidly worse until he was in a critical condition. It was then decided to send for a homœopathic physician and the writer was summoned. Among other symptoms the patient was sensitive to the slightest jarring of the bed, the shock of which was referred to the perineum. This directed attention to Belladonna and the fever, pulse, and other symptoms being found under that remedy it was given with the most gratifying result and the patient recovered.

To return to the enumeration of the characteristics of Belladonna, in headaches a tight bandage around the head relieves.

Patients with congestion to the head bore the head deeply into the pillows and roll the head from side to side. Children suffering from cholera infantum, bore the head so deeply into the pillows that when they are lifted from the bed a deep hole of the general shape of the head remains in the pillow.

*Bearing down in the abdomen* as if everything would issue through the vulva is another prime characteristic of Belladonna. Other remedies have the symptom, but none have it with the same positiveness as Belladonna.

*Desire for acids* is one of the key-notes or characteristics of Belladonna. Under such circumstances the indulgence of the taste for acids is apt to suspend the action of the remedy.

*Aggravation from three o'clock P. M. to three o'clock A. M.* is the characteristic time of Belladonna.

A catarrhal fever caused by taking cold was cured by Belladonna with magical quickness by observing the time when the fever, delirium, and other symptoms came on, and when they remitted.

Belladonna has *convulsive motions or spasms of the muscles of the face, mouth, and throat.* It has chalky stools, like Calcarea, and incontinence of urine from eating sugar and sweets.

Belladonna pains *come on suddenly and as suddenly disappear.* This is one of the great indications which should at once direct attention to this remedy.

Belladonna has been used successfully to cure hernia.

Dr. C. Carleton Smith once cured such a case which he afterward related to Dr. Lippe, on which occasion the writer of this editorial was present. The patient was a workingman who had ruptured himself by falling from a height. The hernia was very large and exquisitely sensitive to touch, and the pain so intense as to induce delirium. The delirium was low and muttering. There was present a colic which, in his lucid intervals, the patient described as a sense of grasping with the finger-nails at the umbilicus. The whole abdomen was tender to the touch, and there was green watery vomiting.

Belladonna was administered, and the next morning the hernia had disappeared without any manipulation whatever.

While all the indications given in this article are perfectly well known to Hahnemannians, and used by them every day, there is a considerable number of people in the profession whose ideas are more or less undefined about the sphere of action of each of these drugs, and who constantly alternate them when uncertain of the right remedy. To all such prescribers this article is especially commended.

The comparisons are derived mainly from Dr. Lippe's lectures and from private conversations with him. They show how clearly he had arranged in his own mind their respective pathogeneses. His knowledge of the other remedies constituting the polychrests of the materia medica was equally well defined, and thus we have the secret of his remarkable success in selecting the right remedy, and thus promptly "healing the sick" as he constantly expressed it.

The lesson his example taught ought to be learned by that great majority of the profession who believe in abolishing the blind adherence to law in prescribing, and in its place practicing according to rationalism, to theory, and to empiricism under the mistaken impression that they are advocating a broad-minded liberalism.

